# Histological Evaluation of the Biocompatibility of Polyurea Crosslinked Silica Aerogel Implants in a Rat Model: A Pilot Study

**DOI:** 10.1371/journal.pone.0050686

**Published:** 2012-12-12

**Authors:** Firouzeh Sabri, John D. Boughter Jr, David Gerth, Omar Skalli, Thien-Chuong N. Phung, George-Rudolph M. Tamula, Nicholas Leventis

**Affiliations:** 1 Department of Physics, University of Memphis, Memphis, Tennessee, United States of America; 2 Department of Anatomy and Neurobiology, UTHSC, Memphis, Tennessee, United States of America; 3 Department of Otolaryngology Head and Neck Surgery, UTHSC, Memphis, Tennessee, United States of America; 4 Department of Biological Sciences, University of Memphis, Memphis, Tennessee, United States of America; 5 Department of Chemistry, Missouri University of Science and Technology, Rolla, Missouri, United States of America; Florida International University, United States of America

## Abstract

**Background:**

Aerogels are a versatile group of nanostructured/nanoporous materials with physical and chemical properties that can be adjusted to suit the application of interest. In terms of biomedical applications, aerogels are particularly suitable for implants such as membranes, tissue growth scaffolds, and nerve regeneration and guidance inserts. The mesoporous nature of aerogels can also be used for diffusion based release of drugs that are loaded during the drying stage of the material. From the variety of aerogels polyurea crosslinked silica aerogels have the most potential for future biomedical applications and are explored here.

**Methodology:**

This study assessed the short and long term biocompatibility of polyurea crosslinked silica aerogel implants in a Sprague-Dawley rat model. Implants were inserted at two different locations a) subcutaneously (SC), at the dorsum and b) intramuscularly (IM), between the gluteus maximus and biceps femoris of the left hind extremity. Nearby muscle and other internal organs were evaluated histologically for inflammation, tissue damage, fibrosis and movement (travel) of implant.

**Conclusion/Significance:**

In general polyurea crosslinked silica aerogel (PCSA) was well tolerated as a subcutaneous and an intramuscular implant in the Sprague-Dawley rat with a maximum incubation time of twenty months. In some cases a thin fibrous capsule surrounded the aerogel implant and was interpreted as a normal response to foreign material. No noticeable toxicity was found in the tissues surrounding the implants nor in distant organs. Comparison was made with control rats without any implants inserted, and animals with suture material present. No obvious or noticeable changes were sustained by the implants at either location. Careful necropsy and tissue histology showed age-related changes only. An effective sterilization technique for PCSA implants as well as staining and sectioning protocol has been established. These studies further support the notion that silica-based aerogels could be useful as biomaterials.

## Introduction

Porous biocompatible materials have received particular attention in recent years for a broad range of applications. From filters and prostheses to scaffolds for tissue engineering, porous biomaterials have been under constant development and improvement for biological and biomedical applications [1], [2], [3], [4], [5], [6], [7]. Complications such as poor mechanical performances, batch-to-batch purity variations, and large pore sizes (µm –range) have limited the extent of use of naturally occurring biomaterials [8] even though they most closely simulate the native cellular environment.

Large pore sizes restrict the use of this class of porous materials to whole cell penetration and tissue infiltration applications only, making them unsuitable for drug delivery and protein loading applications where the physical size of the entities of interest are on the order of 10 s of nanometers rather than microns [9], [10]. Studies have also shown that cellular uptake of drugs is more efficient at the nm scale level [11] emphasizing the need for nm-scale porous materials and membranes as ideal tools for drug delivery. The cell size of porous biomaterials also plays an important role in the formation of scar tissue and fibrosis where major effort is invested to minimize these formations. There is evidence to suggest that closely spaced nanometer-sized pores prevent formation of extensive fibrous connective and scar tissue and in fact promotes superior tissue integration [12], [13]. Additionally, from a mechanical behavior point of view stress concentration greatly depends on pore size and is proven to be significantly less for materials with smaller pore diameters such as mesoporous materials (pore diameter<50 nm) [14], [15].

Aerogels are nanostructured, open-mesoporous (pore diameter<50 nm) ultra low-density lightweight materials with a high surface-to-volume ratio [16] and tunable surface and bulk properties allowing for control over potentially key parameters such as surface wettability, density, opacity, pore size and shape to name a few [17]. Of particular interest to biological applications are the polyurea crosslinked silica aerogels (PCSA) where significant mechanical strength has been accomplished by covalent crosslinking of the skeletal nanoparticles with polyurea without significant compromise of the porosity and the low bulk density of the native material [18], [19]. The biocompatibility of silica particles has been widely explored and has shown a great deal of promise and compatibility with living matter [20], [21], [22], [23]. Drug delivery by porous silica has also been explored and offers great promise [24], [25]. At the cellular level, recent *in vitro* studies on PCSA has shown good biocompatibility of this material [26], [27], [28] and the ability to manipulate the growth of neurons on the PCSA surface with the aid of a laminin layer [26].

The versatility of sol-gel method for material preparation allows for doping and pigmenting techniques to be incorporated at the synthesis stage of aerogels with relative ease [29], [30]. Previous investigations of pigmenting PCSA demonstrated a stable and non-leaching chemistry while also strengthening the mechanically properties and retaining the porosity of the aerogels [31]. This can be of significant interest for biomedical applications since the majority of polymeric implants used today are translucent or transparent. Post-surgical identification of a colored implant may be easier.

In this work, we report the first *in vivo* assessment of the biocompatibility of both clear and pigmented PCSA implants in a rat model sterilized following an ethylene oxide (EtO) sterilization protocol. The presence of the implants did not result in any morbidity. In addition, histological evaluation of the tissues surrounding the implants as well as distant organ sites did not demonstrate inflammation or substantial tissue damage. The results of the PCSA implant study were compared to three control groups: 1) Suture material present (peripheral nerve transection followed by suture repair), 2) sham surgery, and 3) no surgery. Results presented in this study demonstrate the biocompatibility of PCSA thereby supporting the notion that these materials could be useful for biomedical applications.

## Materials and Methods

### Preparation of clear and pigment-doped aerogels

Pigmented and clear polyurea crosslinked silica aerogels were synthesized according to our previously described formulation [30]. The aerogel implants were cut and roughly shaped from the bulk material using a diamond tipped abrasive disk mounted in a rotary tool. The samples were then polished by hand to a final size of 5×2×2 mm^3^ for the back muscle study and 5×2×2 mm^3^ for the deep muscle study. The final stage of implant preparation was exposure to a small vibratory tumbler containing aluminum oxide grinding media (approximately 200 mesh), and processed for about 2 days. The surface contact angle of these aerogels was measured to be around 45° and a density of 0.4 g/cm^3^ for clear and 0.5 g/cm^3^ for pigmented samples was measured. All implants were sterilized for 24 hrs by a standard ethylene oxide sterilization process in an Amprolene system (Anderson Products) prior to surgery.

### Surgical procedure(s)

All surgical procedures were performed on male or female Sprague-Dawley rats weighing 200–300 gr. Rats were anesthetized 15 min prior to surgery via an initial intramuscular injection of Telazol (0.03–0.05 ml at 0.3–0.5 mg/kg), followed by isoflurane inhalation at 1–2%. During the post-surgery recovery period animals were not restrained and were allowed to continue with their normal grooming routine. They were fed a routine diet and kept under close observation for signs of infection or abnormal behavior. Rats were euthanized post implant recovery by an overdose of carbon dioxide. This study was approved by the Animal Care and Use Committee at the University of Memphis.

### Surgical group 1: Subcutaneous Implant insertion (n = 4)

**Figure 1:**
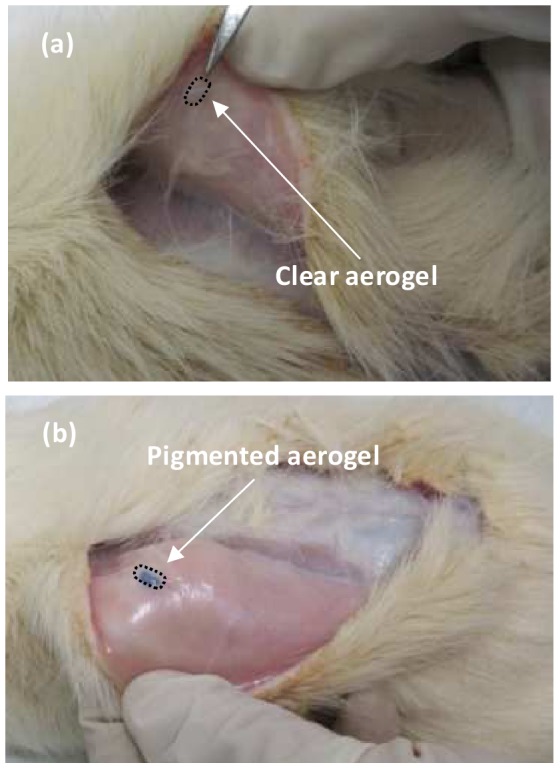
Subcutaneous implant retrieval (surgery site 1). Optical images of (a) clear and (b) pigmented aerogel implants being retrieved after twenty months of subcutaneous incubation in Sprague-Dawley rats. Arrows indicate the embedded implants. A mild fibrosis can be observed for both pigmented and clear PCSA implants. The pigmented aerogel samples were much easier to identify compared to the clear implants.

An area on the back, approximately 3 cm^2^, was shaved and a 1 cm incision was made with a scalpel. One pigmented and one clear implant was inserted subcutaneously in the back of rats (total of 8 implants). Samples were inserted and immobilized without the use of any sutures, adhesives, or staples. Figure 1 shows clear (Figure 1a) and pigmented (Figure 1b) SC PCSA implants after incubation period, prior to extraction.

### Surgical group 2: Intramuscular implant insertion (n = 2)

The left hind limb of rats were abducted and shaved. Under a dissecting microscope, a transverse incision was made through the skin of the limb halfway between the iliac crest and the femur's articulation with the tibia. A self retracting retainer was replaced, and dissection was carried down between the biceps femoris and gluteus muscle until the sciatic nerve was identified. The implant was positioned between the muscle and the sciatic nerve such that the nerve was in direct contact with the surface of the implant. No adhesive or sutures were used for immobilizing the aerogel implant. The skin was then closed with staples and the animal was then allowed to emerge from anesthesia.

### Surgical group 3: Suture repair (n = 2)

The sciatic nerve branches of rats were exposed and transected sharply as described above. The nerve segments were then coapted using standard epineural suture technique with interrupted 9-0 nylon.

### Surgical group 4: Sham surgery (n = 1)

The sciatic nerve of rats was exposed and severed again sharply, similar to the method used for suture repair but after severance, nerve endings were abandoned and no coaptation was attempted.

### Implant and tissue retrieval

At time of sacrifice each animal was weighed and its weight recorded. All animals under investigation in this study continued to gain weight steadily throughout the implant incubation period at the same rate as the control groups. At appropriate recovery time points previous incisions were reopened and the aerogel implant was identified and removed with a cuff of the surrounding tissue intact for further analysis. At two weeks, four of the subcutaneously implanted clear and pigmented PCSA samples (surgical group 1) were removed for analysis. At twenty months four more subcutaneously inserted clear and pigmented PCSA samples (surgical group 1) were removed along with vital organs. Finally, at seven months time point PCSA implants and vital organs were removed from surgical group 2. For the sake of comparison tissue samples from the surgery sites as well as vital organs were removed from surgical groups 2, 3, and 4 at the same time point. Post surgery, animals were anesthetized with inhaled agent and sacrificed at each time point with an overdose of carbon dioxide. All tissue samples were transferred to jars containing 10% formaldehyde.

### Histological staining and examination

Immediately after dissection, tissues containing implants as well as tissues from distant organs were fixed for at least 18 hrs in 10% formaldehyde. Tissue segments obtained from organs without implant were embedded in paraffin and the sections obtained from these paraffin blocs were stained by hematoxylin eosin (H&E) following routine procedures.

**Figure 2:**
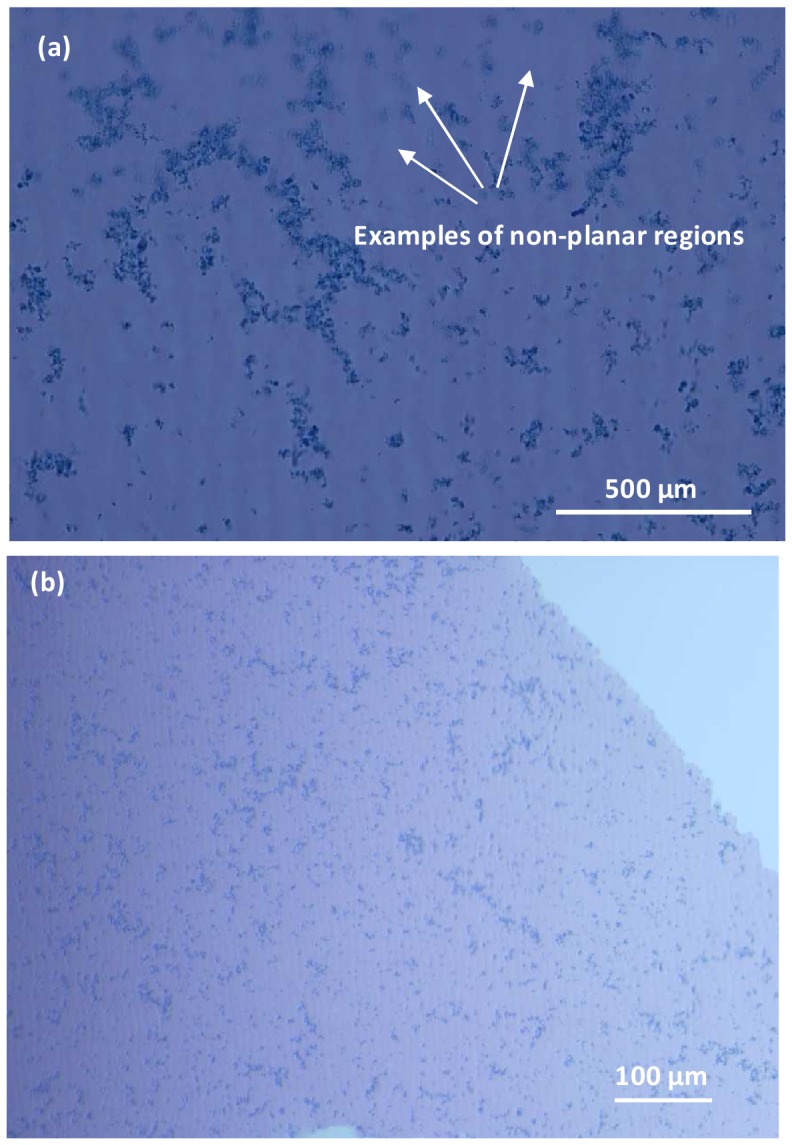
Stain uptake by PCSA sections. H&E stain uptake by 5 µm thick sections of (a) clear and (b) pigmented aerogel samples. The out-of-focus regions of the image (indicated by arrows) reflect the three dimensional nature of the aerogel material. While the pores cannot be visualized by a light microscope, the darker regions are interpreted as openings to the three dimensional porous structure. Images were taken with an upright Nikon Eclipse 800 microscope. Striations noticed in (a) are attributed to sectioning artifacts.

Initial experiments revealed that adhesion of sectioned PCSA aerogel implants to glass slides was challenging. Two protocols were tested to overcome this difficulty. First, implant containing tissue samples were embedded in polymethylmethacrylate (PMMA) and the sections obtained from these blocs were mounted onto plastic slides for staining. This resulted in satisfactory adhesion of tissue/aerogel samples onto the slides but the refractive index of the plastic hindered optimal light microscopy observations. In the second protocol, the implant containing tissue sections were embedded in paraffin following standard procedures. Sections of 5 µm thickness were obtained from the paraffin blocks and various functionalized glass slide surfaces were tested with respect to their ability to bind the implant present in the tissue sections. Slides coated with silane (Electron Microscopy Sciences), poly-L-lysine (Electron Microscopy Sciences), or super-frost coating (Thermo Fisher Scientific) were unsatisfactory as the aerogel implants detached readily early during the staining process. In contrast, the PCSA implant material adhered through the whole staining and mounting procedure on glass slides coated with egg albumin (Newcomers Supply). Sections containing the implant were stained with H&E. Double blinded tissue section evaluations were performed by a pathologist who, in the case of sections without implants, was unaware of whether or not the tissue examined was obtained from an animal with or without implants. Figure 2 shows the effect of the staining protocol on the clear (Figure 2a) and pigmented (Figure 2b) control PCSA sections. It can be seen that the PCSA alone does uptake the stain to a certain extent. Striations seen in the image are considered to be staining artifacts and do not reflect PCSA surface morphology. At times, sectioning the paraffin-embedded tissue-implant samples was difficult due to the hardness of the PCSA implant and several attempts had to be made in order to create a uniform section.

## Results and Discussion

Implant incubation times were chosen based on the three contact duration categories that biomaterials and medical devices are recommended for testing namely 1) Limited (<24 hrs), 2) Prolonged (>24 hrs and <30 days) and 3) Permanent (>30 days) [32]. Aerogel-based biomaterials are expected to serve under classifications 2 and 3 and incubation periods were chosen accordingly

Both clear and pigmented PCSA samples were implanted a) subcutaneously (SC) at the dorsum and b) intramuscularly (IM) between the gluteus maximus and biceps femoris of the left hind extremity in Sprague–Dawley rats. Subcutaneous implants were removed at two weeks and twenty month time points while IM implants were extracted after seven months of incubation.

### Implant Condition

The physical appearance and size of the recovered implants was not altered as a result of short or long term *in vivo* incubation. Macroscopic and microscopic (optical) evaluation of implants showed no signs of erosion, surface deterioration, or fragmenting. Additionally, the implants inserted at the different locations did not appear to have traveled from their original location even though no immobilization (sutures, adhesives, etc) was used. This is particularly surprising in the case of the implants inserted in the hind leg, since the animal had continuous motion and grooming ability and its motion was not restricted during the recovery period. We attribute this to the low density and light weight nature of the aerogel implant. It is likely that the nanoporous/mesoporous and three dimensional nature of the surface of the aerogel implants created anchoring sites between the nearby tissue and the surface of the PCSA implant.

### Inflammation

In general, irritation and inflammation of the tissue that is in direct contact with implants is a concern [33]. In this case however, no significant inflammation was observed for any of the time points, at the interface of the aerogel implants and the nearby tissue for both SC and IM surgery types. The amount of inflammation (according to pathologists report) was categorized as mild in all cases such that the amount of inflammation observed for PCSA implants was comparable to the amount of inflammation observed for biocompatible materials particularly surgical steel and nylon sutures (Tables 1 and 2). This is likely due to the nanoporous/mesoporous nature of the aerogel implant surface that while it is highly non-uniform at the nanometer scale, to the large muscle cells nearby it presents itself smooth and as a result causes minimum irritation and inflammation. A mild inflammation is expected for all foreign materials including biomaterials and is recognized as part of the body's foreign body response [34]. The response seen in this study to the PCSA implants is identified as a normal reaction.

**Table 1:** Summary of short and long term effect of subcutaneous aerogel implant on local tissue.

Incubation PeriodResponse	Two weeks		Twenty months	
	Clear	Pigmented	Clear	Pigmented
Inflammation	Mild	Mild	Mild	Mild
Fibrosis	Mild	Mild	Mild	Mild
Infection	None	None	None	None
Implant travel	None	None	None	None
Age of rat at time of extraction	∼3 months		∼2 years	

Slides from both control and implant-containing rats were reviewed blindly by pathologist, specifically looking for signs of inflammation, infection, fibrosis, and implant travel.

**Table 2:** Summary of the effect of intramuscular aerogel implant on local tissue.

Seven month period				
ProcedureResponse	Sham	Suture	Implant-clear	Implant-pigmented
Inflammation	Mild	Mild	Mild	Mild
Fibrosis	Mild	Mild	Mild	Mild
Infection	None	None	None	None
Age of rat at time of extraction	9–10 months old			

Slides from both control and implant-containing rats were reviewed blindly by pathologist, specifically looking for signs of inflammation, infection, and fibrosis.

### Fibrosis

**Figure 3:**
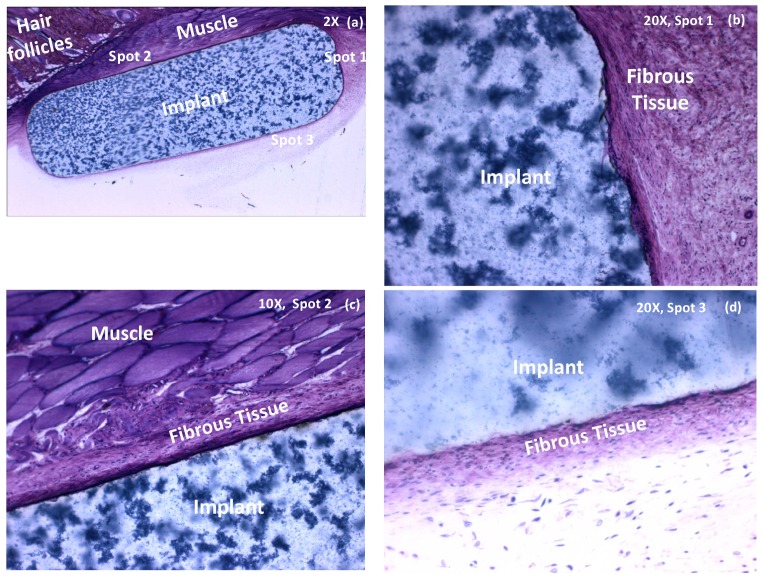
Histological evaluation of the short-term effect of subcutaneous PCSA implants on nearby tissue. Histology of pigmented aerogel implant extracted after two weeks and stained with methylene blue/basic fucshin. (a) All of the aerogel implant at X 2 magnification, (b) X 20 magnification for spot 1, (c) X 20 magnification of spot 2 and (d) X 10 magnifications of spot 3. A mild fibrosis is observed but no inflammation. Images were taken with an Olympus BX51 microscope.

In general, the biocompatibility of novel biomaterials with tissue is evaluated based on the *in vivo* inflammatory responses as well as the fibrosis formed around the implant [35]. Fibrous capsule formation is a well-established reaction to implanted biomaterials and is recognized as the end stage of the foreign body reaction [34]. In this study a mild fibrosis and capsule formation was observed for almost all of aerogel implant-tissue interfaces. Figure 3 shows the SC PCSA-tissue interface behavior for a pigmented sample. In Figure 3a the whole implant is imaged while Figures 3b, 3c, and 3d show high magnification images of interface and the fibrous layer formed around the implant for spots 1, 2, and 3 respectively as identified on the image (Figure 3a).

**Figure 4:**
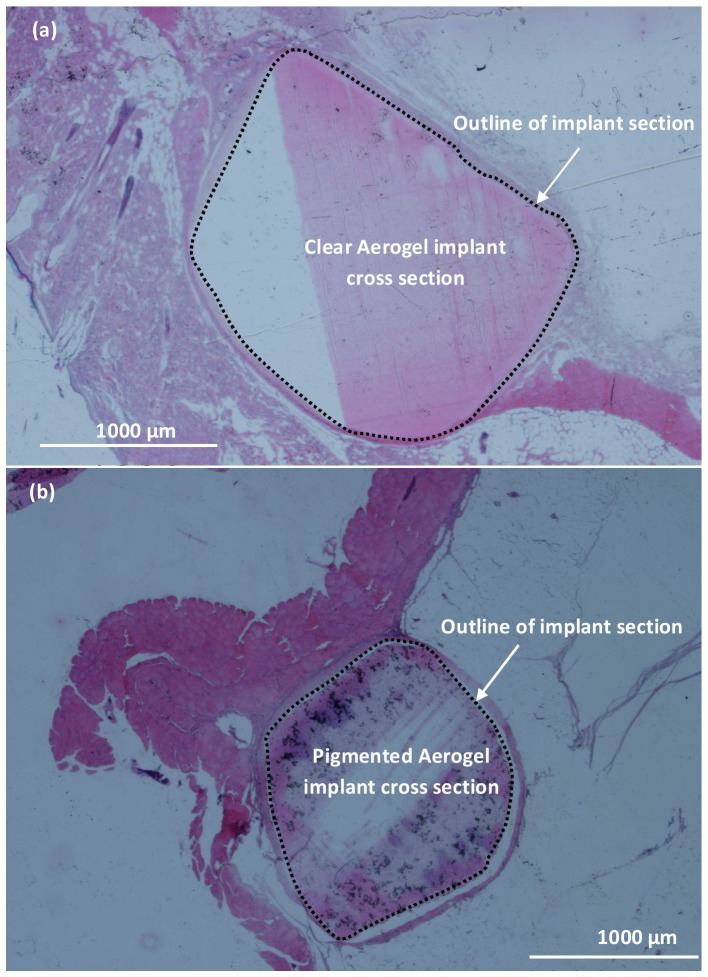
Histological evaluation of the long-term effect of subcutaneous PCSA implants on nearby tissue. Histology of (a) clear and (b) pigmented aerogel implant extracted after seventeen months of subcutaneous incubation in Sprague-Dawley rats and stained with H&E. A mild fibrosis is observed but no inflammation. The dotted line outlines the boundary between PCSA and nearby tissue.

In Figure 4 example cross sectional images of the SC implant insertion after twenty months of incubation are shown for both clear (Figure 4a) and pigmented (Figure 4b) PCSA samples. The dotted line in each case outlines the boundary between the aerogel and the nearby tissue. Some staining of the PCSA section can be seen in each case. The thickness of the fibrous layer for the SC surgery, around the clear and pigmented aerogel implants between two weeks (Figure 3) and twenty months (sample images shown in Figure 4) time points did not show a significant difference. In all cases native cellular tissue had grown up to the implant surfaces. It is not clear from the tests performed here if any tissue had grown into the aerogel pores and interstices. From the histological evaluations it was concluded that the fibrosis around the pigmented implants wasn't significantly greater than fibrosis around clear implants. Table 1 summarizes these results.

**Figure 5:**
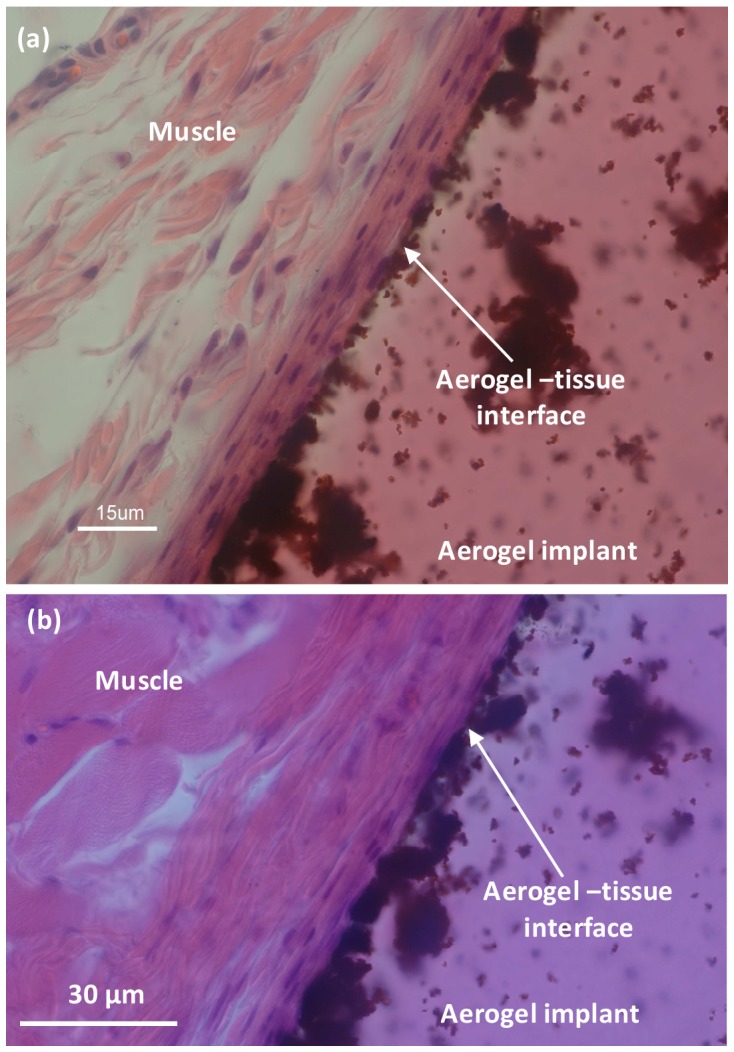
High magnification images of aerogel-muscle interface, intramuscular implantation. Histology of (a) clear and (b) pigmented aerogel implants extracted after seven months of IM incubation. At the interface between aerogel and muscle a mild fibrosis is observed but no inflammation. Darker regions seen on the aerogel side are associated with the three dimensional, no-planar structure of aerogel. Images were taken with a Nikon Eclipse 800 microscope.

Next, the fibrous layer formed around biocompatible materials such as surgical sutures and surgical tools (images not shown) were compared with the fibrous layer formed around PCSA implants inserted intramuscularly. High magnification images of the interface of clear (Figure 5a) and pigmented (Figure 5b) PCSA with nearby tissue extracted after seven months of IM incubation was studied and results are summarized in Table 2. Fibrosis observed in all cases was classified as “mild” and was in agreement with the amount of fibrosis observed at the surgery site for suture and sham surgery procedures. Again, cellular tissue had grown at least up to the implant and perhaps growth has also occurred into the structure of the aerogels although, no specific tests were performed to confirm this.

### Infection

No infection was observed in any of the implant or control conditions, at any of the time points, suggesting that ethylene oxide sterilisation is an effective method for sterilising PCSA implants.

### Toxicity

Toxicity of novel biomaterials is often evaluated by monitoring the response of vital organs distant to the implant. In particular organs such as spleen, lung, heart, kidney, and intestine must be evaluated histopathologicaly [36] for any signs of abnormal responses. In Table 3 the long term effect of aerogels' presence in the system of Sprague-Dawley rats has been summarized. The evaluation of organs removed from animals with 1) suture surgery, 2) no surgery (control), and 3) sham surgery was classified as unremarkable. The organs removed from animals carrying an aerogel implant of some kind were indistinguishable from those previously mentioned. This indicates that in the initial study, no noticeable systemic reaction or immune response has been triggered by the presence of the aerogel implants.

**Table 3:** Summary of long term effect of intramuscular and subcutaneous aerogel implants on organs.

ProcedureOrgan	Sham surgery	Suture surgery	Control (no surgery)	Pigmented/clear aerogel implant-SC	Pigmented/clear aerogel implant-IM
Spleen	Unremarkable	Unremarkable	Unremarkable	Undistinguishable	Undistinguishable
Lung	Unremarkable	Unremarkable	Unremarkable	Undistinguishable	Undistinguishable
Heart	Unremarkable	Unremarkable	Unremarkable	Undistinguishable	Undistinguishable
Kidney	Unremarkable	Unremarkable	Unremarkable	Undistinguishable	Undistinguishable
Intestine	Unremarkable	Unremarkable	Unremarkable	NA	NA

## Summary and Conclusion

This study involves the utilization of *in vivo* tests to determine the general biocompatibility of PCSA as a biomaterial. Double blinded reviews by pathologists showed no statistical difference between tissue samples collected from different surgical groups suggesting tolerance and biocompatibility of PCSA. Early *in vivo* assessment of tissue compatibility presented here can be used to influence the design criteria of future PCSA-based medical devices.

Ethylene oxide sterilization has proven to be an effective method for sterilizing aerogel implants. The *in vivo* studies performed here on a small group of Sprague-Dawley rats have demonstrated biocompatibility of polyurea crosslinked silica aerogels over a maximum of twenty month incubation period. Fibrosis observed was at the normal level that would be observed with any foreign object including sutures. Although this work was performed on a small group of animals, it provides the basis for continuation of the study on a larger group of animals and shows promise for PCSA as a biomaterial. The nanometer scale roughness of the aerogel surface seems to play a significant role in the limited range of motion and travel of the implant from the surgery site therefore, eliminating the need for sutures. The results of our *in vivo* experiments suggest the need for further careful study to better understand the fundamental processes involved in the interaction between this specific type of material and the living body.
